# Supplementary material for: Are carbon emissions trading and green financial instruments synergistic? -Comprehensive quantitative research based on content analysis
Source: PLoS One. 2024 Mar 7;19(3):e0298601. doi: 10.1371/journal.pone.0298601 (PMC10919601; doi:10.1371/journal.pone.0298601)
Supplement: S1 Data — (ZIP) [file pone.0298601.s001.zip › Data/地级市Z_F工作报告.zip!/─┌├╔╣┼/│α╖σ╩╨/└┤╫╘╣1⁄2╓┌║┼í╛┬φ┐╦╩2╛▌═°í┐_2015.docx]

各位代表：

　　现在，我代表市人民政府，向大会报告工作，请予审议，并请市政协委员和其他列席人员提出意见。

　　一、2014年工作回顾

　　刚刚过去的一年，是我市经济社会发展负重前行的一年。面对复杂多变的外部环境和经济下行的内在压力，市政府紧紧依靠全市人民，认真贯彻党的十八大、十八届三中、四中全会精神，全面落实习近平总书记视察内蒙古、李克强总理视察赤峰的重要讲话精神，按照自治区8337发展思路和市委六届五次全委会安排部署，主动适应经济发展新常态，牢牢把握工作主动权，持续加力用劲，不断攻坚克难，全市经济社会实现了平稳健康发展。地区生产总值完成1778.4亿元，增长7.9%；公共财政预算收入98亿元，增长7.5%；500万元以上项目固定资产投资1101.3亿元，增长16%；社会消费品零售总额587.4亿元，增长11.6%；城镇和农村牧区常住居民人均可支配收入达到23199元和8114元，分别增长9.7%和11.4%。

　　（一）多措并举应对经济下行压力，产业发展稳中有进。把保工业作为稳增长的首要任务，落实电价补贴和贷款贴息8551.7万元，减免缓税费2.75亿元，推动65户停产半停产企业恢复生产，减少亏损企业36家；全市规模以上工业增加值增长10.7%，冶金产业销售收入保持千亿元以上，3家企业和5个工业园区销售收入超过百亿元。产业结构调整取得积极进展，冶金、能源产业占比下降1.4个百分点。稳步发展农牧业，新增高效节水种植面积162万亩，粮食产量在大旱之年保持百亿斤以上；牧业年度牲畜存栏2215万头只，连续9年居自治区首位；新培育国家和自治区级农畜产品品牌31个。加快推进服务业提质增量，万达广场、新天地广场二期、金融物流港一期、雨润农副产品物流配送交易中心等一批重点商贸项目建成运营，新增商业面积198万平方米。赤峰保税物流中心封关运营，现代物流业发展迈上新的台阶。推进一批特色旅游景区和四季旅游品牌建设，全年接待游客1139万人次，实现旅游收入180亿元，分别增长20.9%和23.9%。新增金融机构及营业网点18家，中信银行入驻我市，宁城农商行挂牌运营；全市贷款余额1048.1亿元，增长21.8%，高于存款增速14个百分点。

　　（二）全力抓好项目建设，发展后劲进一步提升。燕京啤酒扩建、草原水泥、浩克新能源汽车、拓佳液晶显示屏等一批重点项目建成试产。大地云天缓控释肥、中电投热电联产等一批投资10亿元以上新项目开工建设。完成交通、水利、电力等基础设施建设投资88.9亿元，一批学校、医院、文体场馆等社会事业项目建成投入使用。紧紧围绕落实李克强总理两次视察我市的重要讲话精神，推动赤峰至京沈高铁喀左站项目建议书获得国家批复；建设服务京津冀地区清洁能源输出基地、元宝山电厂接入冀北电网、克旗120万千瓦抽水蓄能电站、防沙治沙和湿地保护项目获得国家明确支持；东台子水库项目进入国家审批环节，经棚至承德高速公路可研报告通过自治区初审；总理关注的翁牛特旗系列事项得到全面落实；铁南棚户区改造工程已争取上级资金和银行贷款41亿元，房屋征收、安置房基础及配套服务建设全面展开。

　　（三）全面深化改革开放，发展活力不断增强。工商登记制度改革启动实施，新登记企业增长89.2%，注册资本增长303.5%。农村牧区土地和草牧场经营权流转规模突破800万亩。全面放开县城和建制镇人口落户限制，大幅精简户籍办理手续，户籍迁转更加方便快捷。实施初中升高中五统一考试招生制度和幼儿划片、摇号入园办法，实现了公平录取、阳光招生。落实单独两孩政策，四个旗县启动县级公立医院综合改革试点工作。通过市场化运作举办了第九届红山文化节。推进政府机构改革，市政府减少工作部门5个。市本级取消和下放行政审批事项158项，精简比例60.3%。市县两级政府公开了财政预决算，72个市直部门公开了年度预算。推进公共资源交易市场建设，市级交易平台完成交易额109亿元。赤锡合作上升为自治区发展战略，昭乌达老工业区纳入国家搬迁改造试点，中关村海淀园赤峰分园落户赤峰经济开发区。全年承接北京市非资源型产业转移项目39个，引进国内市外500万元以上项目资金516亿元，增长11.4%。

　　（四）注重统筹城乡协调发展，城乡面貌持续改善。中心城市框架进一步拉大，建成区面积105平方公里，人口达到百万，城市功能日趋完善。新建改造供热、供气、给排水管网160公里，新增供热能力360万平方米，建成10万吨再生水利用工程；机场快速路、友谊大桥、大板桥建成通车，解放街西通和广场路、银河路改造工程完成；集中开展了拆违拆临、垃圾清理、打击黑车、整治马路市场和露天烧烤等专项行动，城市环境更加干净整洁。各旗县统筹推进新老城区、建制镇和新农村新牧区协调发展，城镇基础设施建设完成投资39亿元。全市开通延伸公交线路30条，新增公交车46辆。民航进出港旅客达到88.1万人次，增长35%。新建商品住宅361万平方米，开工保障性住房1.6万套，实施老旧小区改造275万平方米，完成农村牧区危房改造2.05万户，接管物业弃管楼65万平方米，城乡居民居住条件进一步改善。投资49.5亿元，完成498个重点嘎查村十个全覆盖工程建设任务。

　　（五）全面打响环境治理攻坚战，生态环境整体好转。实施重点行业脱硫脱硝除尘改造项目80个，淘汰取缔燃煤锅炉388台，拆除中心城区燃煤炉灶1.3万台。中心城市主要路段实施黄标车限时禁行，淘汰报废黄标车和老旧车3.9万辆。采取停产限产、秸秆禁烧、公车停驶等措施，圆满完成APEC会议期间空气质量保障任务。全年淘汰60万吨水泥、7万吨铜铅冶炼、6万吨制革落后产能。45家重金属企业实现24小时在线监控。出台地下水管理办法，7个城镇污水处理厂完成提标改造，12个辽河流域水污染治理项目竣工投运。投资1.2亿元，实施了平庄矿区等6个矿山地质环境治理工程。投资12.8亿元，新增营造林101.2万亩，完成重点区域绿化24.2万亩。乌兰坝自然保护区晋升为国家级自然保护区。

　　（六）千方百计增进人民福祉，社会大局保持和谐稳定。全市民生支出277.3亿元，占公共财政预算支出的76.9%。城镇新增就业3.2万人，登记失业率控制在4%以内，农村牧区劳动力转移就业70万人。蒙东云计算中心成为全国创业孵化示范基地及国家级科技企业孵化器。城镇职工和新农合住院报销封顶线提到30万元，城镇居民基本医疗保险最高支付限额提到12万元。城乡低保标准年分别提高252元和197元。新增各类养老服务机构62所，12349社区公共服务平台覆盖城关镇社区。85.3万户低收入农牧户取暖用煤补贴发放到位，3187名低保家庭大学生得到资助。新增通油路的嘎查村162个，硬化嘎查村街路2200公里。解决30万人饮水安全问题，14万贫困人口实现稳定脱贫。赤峰·中国北方农业科技成果博览会进入全国农高会序列，组建了赤峰有色金属工业技术研究院。全市高考本科上线率提高8个百分点。内蒙古交通职业技术学院被评为全国职教先进单位，赤峰工业职业技术学院成为国家高技能人才培训基地。投资1.2亿元，完善了市县乡村四级文化设施，开展文化惠民演出1100多场次，组建了赤峰话剧团和民乐团。全市一体化卫生信息系统搭建完成。全国文明城市创建工作取得阶段性成果，市民文明素质和城市文明程度进一步提高。成功承办自治区第十三届运动会，获得金牌、奖牌和总分三个第一。圆满完成第三次经济普查工作任务。实施信访百日攻坚专项行动，化解信访积案1636件。组织开展打黑除恶、警灯行动、食品药品安全隐患大排查、安全生产打非治违等专项行动，有效遏制了重特大安全事故和恶性案件的发生。国防建设、双拥共建、民族宗教、外事侨务、妇女儿童、残疾人、气象、地震、人防、档案、慈善等各项工作都取得了新成绩。

　　在推进经济社会发展的同时，我们切实加强政府自身建设，政府的公信力和执行力明显提升。深入开展党的群众路线教育实践活动，严格执行中央八项规定和加强作风建设各项要求，着力整治四风顽疾，市政府会议、文件分别减少16.3%、15.7%，全市收缴封存处置违规公务用车522辆，清理腾退办公用房21万平方米，三公经费较上年压减了11.7%。废止和宣布失效2000年以来市县乡三级红头文件507件。通过规范公共资源交易、实施财政投资项目评审，节约资金27.4亿元。自觉接受人大及其常委会的法律监督、工作监督和政协的民主监督，定期向人大报告工作、向政协通报情况，全年办理人大代表建议159件，政协委员提案185件，满意率87.2%。政务公开、行政监察、审计监督和依法行政工作成效明显，廉政建设和反腐败工作不断加强。

　　各位代表，过去一年我们付出了艰辛努力，取得了来之不易的成绩。这是自治区党委、政府和市委坚强领导的结果，是全市上下群策群力、务实拼搏的结果。在此，我代表市人民政府，向全市广大干部群众，向所有关心、支持、参与赤峰建设和发展的同志们、朋友们，表示衷心的感谢，并致以崇高的敬意！

【分页符】

　　在总结成绩的同时，我们也清醒地看到，经济社会发展中还存在不少困难和问题。主要是：经济下行压力加大，经济发展内生动力不足、创新驱动能力不强，一些企业生产经营仍很困难；主导产业过于依赖资源，结构不合理，传统产业层次低转型慢，新兴产业规模小实力弱，服务业发展相对滞后；生态环境依然十分脆弱，水资源供需矛盾日益突出，发展保障能力亟待提高；群众关心的就业、教育、医疗、住房、食品安全、扶贫攻坚等方面仍存在薄弱环节，征地拆迁、民间借贷、农民工工资拖欠等引发的矛盾纠纷有所增多；一些地区和部门抓落实力度不够，部分干部责任意识和担当意识不强，四风问题仍不同程度存在，作风建设常态化机制有待进一步完善。对此，我们一定采取更加扎实有力的措施认真解决。

　　二、2015年工作安排

　　今年是全面深化改革的攻坚之年，是全面推进依法治市的开局之年，也是收官十二五、谋划十三五的关键之年。在新常态下，我们既面临着宏观形势偏紧、经济下行压力加大、结构矛盾突出、区域竞争加剧等困难和挑战，也面临着全面深化改革红利逐步释放、公共产品投资持续增加、新型城镇化战略加快实施、沿海发达地区产业加速转移等诸多机遇。我们既要看到有利条件和积极因素，又要充分估计可能遇到的困难和问题，坚定信心，趋利避害，顺势而为，奋力开创全市经济社会发展新局面。

　　今年政府工作的总要求是：深入贯彻落实中央、自治区系列重要会议和市委六届六次全委会议精神，坚持稳中求进工作总基调，坚持以提高经济增长质量和效益为中心，准确把握经济发展新常态，统筹推进四个全面战略，更加注重调整结构和转变方式，更加注重深化改革和扩大开放，更加注重统筹城乡和夯实基础，更加注重保护生态和集约资源，更加注重保障民生和促进和谐，努力为十二五发展画上圆满句号。

　　主要预期目标是：地区生产总值增长8%，公共财政预算收入增长6%，固定资产投资增长13%，社会消费品零售总额增长11%，城乡居民收入分别增长10%和11%；城镇登记失业率控制在4%以内，居民消费价格涨幅控制在3%左右，完成自治区下达的各项节能减排任务。提出这样的指标，综合考虑了国家、自治区新常态下的发展大局，考虑了发展需要与可能，兼顾了就业形势、市场状况、资源承载和环境容量等因素，并与完成十二五、启动十三五、全面建成小康社会目标充分衔接，既不盲目追求高指标、高增长，也避免因为换挡造成失速，努力做到调速不减势、增量与提质并行，确保全市经济持续健康发展。

　　实现上述目标，重点要做好以下几个方面工作：

　　（一）同步推进经济总量扩张和提质增效

　　一是狠抓重点项目建设。坚持发展第一要务不动摇，充分发挥投资拉动经济增长的关键作用，把握投资导向，消除投资障碍，创新投融资方式，努力扩大有效投资，保持经济稳定增长。围绕总理视察我市时关注事项，主动向上对接协调，加快实施一批具有全局性、战略性的重大项目。抓好列入自治区调度的60个重点项目建设，确保按计划开工、按序时完成投资。力促富邦10万吨电解铜、澳亚8万头奶牛养殖、凌志7万吨薯条加工、众益糖业5万吨白糖等新项目开工建设，加快实施170万千瓦风电、20万千瓦光伏发电、24个输变电工程、大地云天120万吨缓控释肥、瑞阳6万吨季戊四醇、中新12万锭纺纱、中粮150万口生猪全产业链等项目，推动浩克新能源汽车一期、拓佳液晶显示屏一期、嘉隆新型建材、伊品过瘤胃饲料等项目达产达效。做好审批、征地、环评等项目管理和服务保障工作，提高项目质量。加快推进十三五规划编制，谋划好关系经济社会发展全局的重大政策、措施和项目，为推动科学发展、实现富民强市提供有力支撑。

　　二是推进工业转型升级。围绕五大基地建设调整优化产业结构，进一步提高资源综合利用率和产业精深加工率，加快构建多元发展、多极支撑的现代产业体系。认真落实扶持企业发展的政策措施，加强银企对接、产需衔接、要素保障和生产运行组织。支持各旗县区培育主营业务收入过百亿元产业，鼓励工业企业技改升级、引联重组、开拓市场、延长链条，年内新增规模以上企业30户、亿元以上企业10户。支持传统产业从初级工业品向中高级转化，推进现代煤化工向下游产品生产，有色金属生产加工和装备制造向中高端发展，食品加工向质优、高端消费品延伸。大力发展装备制造、新材料、节能环保、生物医药等战略性新兴产业，积极培育新的经济增长点。推进风险探矿、深部探矿和外围找矿，增强矿产资源的后续保障能力。大力发展信息产业，加强以云计算为重点的信息化基础设施和服务能力建设，推进新型工业化、信息化、城镇化和农牧业现代化同步发展。优化工业园区布局和功能定位，围绕主导产业制定中长期发展规划和产业上下游配套规划，推动原材料前端生产企业和环保压力大的企业退城搬迁。筹措资金20亿元，加大重点园区基础设施建设力度，完善配套服务功能，提高承载能力，吸引更多的企业和项目入驻。加快工业园区提档升级步伐，推动克旗工业园区进入百亿级园区行列，力争赤峰经济开发区进入国家级园区行列。加快实施创新驱动发展战略，推动清洁能源、新型化工、冶金、医药等重点产业技术攻关，培育一批具有自主知识产权的中小型科技企业。新增国家级高新技术企业2家，申报有色金属产业技术创新战略联盟。

　　三是加快推进农牧业现代化。坚定不移地转变农牧业发展方式，从主要追求产量增长和拼资源、拼消耗的粗放经营，向数量质量效益并重、注重提高竞争力、注重农业技术创新、注重可持续的集约发展转变。推进粮安工程和节水增粮行动，农作物播种面积稳定在上年水平，粮食产量保持在百亿斤以上。继续实施 1571工程，大力发展节水型、标准化、规模化种养业。以市场为风向标，推动农牧业由生产导向向消费导向转变，抓好谷子、荞麦、绿豆等优势作物种植，推广赤峰小米、赤峰荞面、赤峰绿豆、昭乌达肉羊等地域品牌，培育市场需求旺盛的绿色有机产品。坚持把产业链、价值链等现代产业组织方式引入农牧业，加快推动传统种养业与食品加工、商贸物流、电商平台、生态旅游等二、三产业融合互动。进一步扶持壮大农牧业龙头企业，健全完善农畜产品市场体系。健全农牧业社会化服务体系，加快培育家庭农牧场、种养大户、农牧民合作社等新型农牧业经营主体，培育新型职业农牧民。推进多种形式适度规模经营，有序流转土地经营权，完成农村土地承包经营权确权登记颁证试点和草原确权承包试点工作。完善农牧业灾害预警预报制度，加强病虫害防治、疫病防控、科技推广体系建设，提高农牧业防灾减灾能力。

【分页符】

　　四是做大做强文化旅游产业。以建设国内一流旅游目的地为目标，加强旅游公共服务设施建设，强化旅游市场监管，进一步提升旅游景区档次和接待服务水平。保护性开发自然景观、地质遗迹和文化遗址，加强地质公园建设，抓好辽上京文化旅游度假区、玉龙沙湖等景区开发，推动道谷南山体育休闲旅游区、敖汉温泉城晋升为国家4A级旅游景区，做好克什克腾国家5A级景区创建工作。通过市场化方式办好各类文化旅游节庆活动，推进文化旅游纪念品开发，强化战略营销和宣传推介，提升赤峰旅游知名度。全年接待游客突破1200万人次，旅游收入达到190亿元。编制完成文化产业发展规划，设立2000万元文化产业专项扶持资金。拓宽文化产业投融资渠道，培育多元化产业主体，扶持特色中小微文化企业发展，形成一批具有核心竞争力的特色文化企业和文化品牌。

　　五是大力发展商贸物流业。依托现代信息技术，加快电子商务推广与应用，建设区域性综合服务平台，提升商贸物流业水平。推动食尚天街、中韩创新经济合作项目尽快启动建设，确保永业广场、万悦广场等商业项目开业运营。制定全市物流业发展规划，出台相关扶持政策，引导物流企业整合资源、加快发展，支持旗县结合本地实际，建设功能完备的物流园区。加快推进赤峰商贸物流城建设，支持红山物流园区拓展发展空间和服务范围，引进第三方物流、冷链物流等新兴业态，推动金融物流港二期、九州通现代医药物流中心等项目年内建成运营。充分发挥保税物流中心作用，加强与口岸、港口对接，深度集聚商贸、物流、信息、金融等服务业群，着力打造赤峰国际陆港。推进新型基层供销合作体系和新网工程建设，加快农村牧区商贸物流业发展。落实好国家和自治区鼓励居民消费的财税、信贷和信用政策，加快培育更多的消费增长点。

　　（二）积极回应人民群众幸福期盼

　　一是推进大气污染防治工程，努力让群众呼吸上新鲜空气。投资12亿元用于大气污染治理。坚守产业政策和环保达标两条底线，利用市场和政策双重机制，倒逼现有企业技改升级、节能减排。加快建设中电投2×30万千瓦热电联产和富龙2×5万千瓦背压机组项目，取缔城市建成区10蒸吨以下燃煤锅炉，确保全市排放油烟的餐饮场所全部安装油烟处理装置。加大机动车尾气治理力度，加速淘汰黄标车和老旧车，严格执行中心城区限时限区域禁放烟花爆竹相关规定。加强建筑工程施工规范化管理，有效控制建筑工地扬尘污染。严格落实《环境保护法》，对各类环境违法行为零容忍和重打击。

　　二是推进城乡安全饮水工程，努力让群众喝上干净水。投资5.2亿元用于改善居民饮水条件。实施三座店水库向中心城区引供水、第二水源地迁移扩建工程，加快雨污分流、城市给排水、再生水管网建设，集中供水管网到达的区域，生活用水一律接入集中供水管网，中水管网到达的区域，除食品、制药等对供水有特殊要求的企业外，工业企业生产用水一律使用中水或地表水，切实减轻地下水开采压力。加快水权统一管理，严格实行中心城区排水许可证制度，足额征收污水处理费。完成中心城区10万吨污水处理项目，实施6.7公里污水截流干管工程。新建农村牧区饮水安全工程467处，解决26.5万农牧民饮水安全问题。加强水源地保护和水质监测，推进重点流域治理，严厉打击偷排偷放行为，切实保障城乡居民用水安全。

　　三是推进食品安全监管工程，努力让群众吃上放心食品。加强食品药品领域日常监管，实行最严格的监管、最严厉的处罚、最严肃的问责，确保人民群众舌尖上的安全。围绕建设绿色农畜产品生产加工输出基地这一目标，全方位建立农畜产品质量安全检验检测和追溯体系，强化从田间到餐桌全过程监管，严把食品安全各道关口。加快农畜产品加工园区建设，解决小作坊多、散、乱、差和监管困难等问题。加强食品质量安全品牌建设，培育更多的驰名商标、著名商标、地理标志证明商标和绿色有机农畜产品品牌。

　　四是推进综合交通畅通工程，努力让群众出行更加便捷。投资98亿元改善市域交通。加快中心城区铁南大街、西大桥、红山大桥、西站大桥、天义桥等20个道路桥梁建设，确保中环路西段开通，打通一批组团连接路和断头路，缓解城市交通拥堵压力。新建一批公共停车场，科学划定临时停车位，加强停车秩序管理，有效解决停车难问题。大力倡导绿色出行，规划建设新老城区沿河自行车专用道。推进中心城区50公里公交服务圈建设，实现赤元客运公交化。加快经锡高速等国省干线公路建设，新建通村油路1000公里。加强道路建设质量监管和农村公路养护工作。开工建设赤峰连接京沈客专快速铁路，推进集通铁路扩能改造和巴新、林白、锦白铁路建设，力争叶赤铁路扩能改造、京通铁路电化改造工程获批。推动玉龙机场迁建和林西支线机场前期工作，完成阿旗通用机场建设。

　　五是推进生态建设和保护工程，努力让群众享受绿色空间。投资12.9亿元用于生态建设。围绕建设京津辽沈地区重要生态安全屏障，加大防沙治沙力度，认真执行草畜平衡制度和封育禁牧措施，落实好草原生态奖补政策，探索发展草原生态畜牧业和林沙草产业的有效途径，切实保护生态环境。全年完成重点生态工程80万亩、重点区域造林绿化20万亩、水土保持综合治理205万亩。大力发展资源节约、环境友好型农牧业，综合施策，减少农牧业投入品过量使用，逐步退出超过资源环境承载能力的生产，推进农牧业废弃物转化利用，切实保护耕地和草原。积极推进编制自然资源资产负债表，开展自然资源离任审计，建立生态环境考核评价体系和责任追究制度，启动排污权有偿使用和交易试点工作。抓好重点区域、重要地段绿化设计，投资4.5亿元，新建城镇园林绿化项目56个。加快国家园林城市创建步伐，推进一环两线三山五河景观系统提升改造工程和锡伯河百公里水利风景观光带工程，进一步提升城市形象和品位。

　　六是推进城市管理和百姓安居工程，努力让群众生活得更舒心。依法执行新版城市总体规划，合理利用城市地上、地下空间，完善公共服务功能，提升居住舒适度和产业承载力。抓好城市专项规划和城乡统筹规划编制，推动中心城市、小城镇和新农村新牧区协调发展。加快实施城镇重要节点和关键部位亮化美化工程，提升城市品位和档次。围绕建设智慧城市，推进信息化与城镇化融合发展，启动中心城市地理信息系统建设，建立基础数据共享的城市数据中心，推动城市管理服务市场化改革，提高城市管理的精细化、智能化水平。投资37亿元用于保障性安居工程建设。筹集公租住房1022套，实施老旧小区改造200万平方米，完成农村牧区危房改造2.1万户。多渠道筹措资金，实施33个棚改工程，改造棚户区11348户，确保铁南棚户区74万平方米安置房全部开工，在建工程主体封顶。推动房地产市场健康发展，全年新开工商品住宅面积350万平方米。

　　（三）全面深化改革开放

　　一是坚持以改革激发活力。积极发展混合所有制经济，大力推广政府和社会资本合作的PPP模式，推动社会资本参与城市基础设施建设运营管理，提高公用事业管理水平。加快组建国有资本运营公司或投资公司，确保交通投融资平台投入运营。推进资源资本化改革，加强统一规范、高效运转的公共资源交易平台建设。落实市场监管体制改革，加强对市场主体监督管理。深化商事登记制度改革，稳步实施先照后证，推进三证合一和工商注册电子化登记管理。组建全市不动产登记管理机构，完成农村牧区集体建设用地使用权确权登记发证工作。严格执行机构限额，控制编制总数，全面完成政府机构改革任务。深化财税体制改革，加强政府债务规模控制和分类管理，规范政府举债行为。扩大政府购买服务范围，实现市县两级政府及部门预决算公开全覆盖，启动县级财政支出管理绩效综合评价试点工作。改进住房公积金提取、使用和监管机制，扩大公积金保障覆盖面。深化文化体制改革，引导优势文化资产引联重组。加快医药卫生体制改革，全面推开县级公立医院综合改革，提升医疗质量和服务水平。加快户籍制度改革，建立居民居住证制度。按照中央和自治区要求，完成公务用车制度改革任务。

　　二是坚持靠开放增强动力。以新思维、新视角谋划推动新常态下的对外开放工作，从注重发挥资源优势向注重发挥区位优势转变。发展飞地经济，积极承接发达地区产业转移，打造承接产业转移示范基地。主动对接国家一带一路战略和京津冀协同发展战略，加快融入环渤海经济圈。抓好园区招商和产业链招商，加快引进高端产业、先进技术和项目资金。加强与驻赤央企合作，推进央地共建园区。抓好外贸进出口基地建设，鼓励有实力的企业参与市外资源开发和产业合作。认真落实自治区向北开放战略，加快发展外向型经济。推进与黑山尼克希奇市、蒙古国后杭盖省缔结友好地区进程。积极引进外埠金融机构来我市设立分支、开展业务，推动兴业银行落户赤峰。积极推广普惠金融业务，发挥金融助推地方经济发展主渠道作用，力争全年贷款余额突破1200亿元。创新投融资方式，培育多层次资本市场，降低社会融资成本，鼓励民间资本发起设立产业投资和股权投资基金，支持有条件的中小企业通过创业板、中小企业板、新三板等进行股权融资和上市融资。推动担保、再担保机构及小额贷款公司规范健康发展，严厉打击非法集资、恶意逃废金融债权等违法行为，维护金融秩序稳定。把引智放到与引资同等重要的位置，大力实施玉龙人才工程，提高人才工作水平。

【分页符】

　　（四）更加注重民生和社会建设

　　一是突出抓好民生改善。进一步优化支出结构，新增财力重点用于民生投入，节约的三公经费全部用于改善民生。深入实施农村牧区十个全覆盖工程，认真落实规划方案，科学调配资源，合理摆布项目，突出各自特色，精心组织施工，加强后续管理维护，切实把这项最大的综合性民生工程抓紧抓实，今年确保高质量完成1156个嘎查村和51个农牧林场建设任务。深入实施创业就业工程，大力推动大众创业和万众创新，突出抓好以高校毕业生就业和化解结构性失业为重点的各类群体就业工作，新建2个小微企业创业园和创业孵化基地。全年城镇新增就业3.1万人，农村牧区劳动力转移就业68.5万人。完善机关事业单位工资制度，调整基本工资标准，落实带薪休假制度。抓好各类社会保险提标扩面工作，落实国家关于机关事业单位养老保险制度改革政策，继续提高企业退休职工和城乡居民基础养老金、优抚对象抚恤、城乡低保对象补助水平。建立完善居家养老为基础、社区养老为依托、机构养老为支撑的养老服务体系，探索城乡互助养老新模式。制定完善扶持民办养老机构的优惠措施，加快建设赤峰老年社区和一批社区老年人日间照料中心、农村互助幸福院。继续做好低收入农牧户取暖补贴发放和低保家庭大学生入学资助工作。深入实施精准扶贫，扎实推进金融扶贫富民工程，强化三到村三到户帮扶措施，筹措资金35亿元，打好扶贫攻坚战，确保14万贫困人口实现稳定脱贫。

　　二是提高社会事业发展水平。承办好2015中国北方农业科技成果博览会。加快基础教育学校标准化建设和薄弱学校改造工程，推动义务教育均衡发展，确保林西县通过国家义务教育均衡发展验收。加强教师队伍建设，提高师德水平和业务能力。加快发展普惠性优质民办幼儿园，新建、改扩建一批公办幼儿园，让更多儿童享受优质学前教育。促进普通高中多样化特色化办学和高等教育内涵式发展，统筹推进民族教育、特殊教育、民办教育发展。加快现代职业教育体系建设，确保赤峰工业职业技术学院通过教育部人才水平评估、内蒙古交通职业技术学院通过自治区示范性高等职业院校建设项目验收。推进市直卫生计生机构建设和旗县区蒙医中医医院能力建设，改造18个乡镇卫生院，新建492个标准化嘎查村卫生室。加强传染病预防控制，健全公共卫生应急管理和预案体系。推进文化强市建设，大力实施文化惠民工程，加大非物质文化遗产保护力度，进一步繁荣文艺创作，促进优秀民族文化传承保护和创新发展。启动赤峰城区历史街区保护工程，开工建设辽上京国家考古遗址公园和二道井子遗址保护馆，推动红山文化遗址、辽代上京城和祖陵遗址申遗工作取得实质进展。深入开展社会主义核心价值观学习教育，继续抓好全国文明城市创建活动，提高公民道德素质和社会文明程度。大力开展全民健身活动，促进群众体育与竞技体育协调发展。抓住我市被列为自治区东部重点足球基地的机遇，推动足球运动广泛开展。

　　三是改进社会治理方式。推进行业诚信建设，发展各类志愿服务组织，加快社会组织去行政化。加强社区居民自治，依法做好村民委员会换届工作。加强行业性、专业性人民调解组织建设，完善人民调解、行政调解、司法调解联动工作体系。严格落实属地管理、分级负责信访工作原则，加强信访工作基层基础建设，及时就地解决各类矛盾，引导群众依法、逐级、理性表达诉求。加强高危行业、重点领域、重要环节的安全生产大检查，严格落实一岗双责制和一票否决制，有效预防和减少各类安全事故。加大劳动执法监察力度，重拳查处克扣拖欠农民工工资等违法行为。深入开展法治宣传教育，深化基层组织和部门、行业依法治理，推进依法治市进程。加快建设覆盖城乡居民的公共法律服务体系，完善法律援助制度，健全司法救助体系。加强网络舆情监管，积极引导和回应社会舆论。加快完善灾害事故和公共事件预警、应急处置体系建设，提高预防和应对能力。以提高群众安全感和满意度为目标，深化平安赤峰建设，加强重点领域基层执法力量，加快公共安全视频监控系统建设，完善立体化社会治安防控体系，严密防范、严厉打击违法犯罪活动。支持国防和军队建设，争创全国双拥模范城八连冠。认真落实党的民族宗教政策，维护团结和谐大好局面。

　　（五）全面加强政府自身建设

　　经济发展进入新常态，政府精神面貌要有新状态。我们要始终保持一种昂扬向上、改革创新的精神状态，始终保持一种公道正派、甘于奉献的思想境界，始终保持一种干就干好、一抓到底的工作韧劲，在困难面前敢闯敢试，在矛盾面前敢抓敢管，在责任面前敢作敢为，着力打造人民满意政府。一是建设法治政府。深入贯彻落实十八届四中全会精神，把依法行政贯穿于政府工作各环节，尽快制定公布行政权力清单。扎实推进政府协商民主建设，依托高校和社会组织建立政府决策咨询论证智库，完善政府法律顾问制度，严格执行重大行政决策程序规定，确保政府决策科学化、民主化。优化整合行政执法力量，切实做到严格规范公正文明执法。二是建设服务政府。巩固党的群众路线教育实践活动成果，继续整治四风顽疾。坚持用刚性制度管权限权，进一步规范投资项目前置审批，加强事中、事后监管，坚决打掉寻租空间。优化审批流程，减少审批环节，加快信息化建设，推广网上并联审批等模式，让企业和群众从简政放权中切实得到实惠和便利。加强电子政务建设，推进各项政务服务更加方便、快捷。三是建设务实政府。自觉践行三严三实，脚踏实地、力戒空谈，大力精简会议和文件，腾出时间精力，多蹲点调研，多下访解难，多上门服务，努力营造让群众和投资者放心、安心、舒心的生产生活环境。健全和完善抓落实机制，说了的坚决做到，定了的坚决执行，部署的强力推进，严肃处理为官不为、懒政怠政行为。四是建设廉洁政府。严格执行中央八项规定，强化公务接待、用车、用房的规范管理，集中清理机关事业单位吃空饷问题，全年三公经费和财政供养人员只减不增，政府收支一律纳入预算并公开，未列入预算的不再追加，最大限度降低行政成本。坚持党风廉政建设和反腐败斗争常抓不懈，加强对重点领域和关键环节的行政监察，大力推进对公共资金、国有资产、国有资源、领导干部履行经济责任情况的审计监督全覆盖，把权力关进笼子里，把政务晒在阳光下。

　　各位代表，我们深知，政府的权力来自人民，政府工作必须置于人民的监督之下。我们一定自觉接受人大及其常委会法律监督和工作监督，自觉接受政协民主监督，自觉接受社会舆论监督，虚心听取各民主党派、工商联、离退休老干部和社会各界人士的建议和意见，主动加强与工青妇等人民团体联系，不断改进政府工作，认真履职尽责，不辜负全市人民的信任和期望。

　　各位代表，新常态孕育新机遇，新机遇催生新发展。尽管我们在前进的道路上有困难、有风险，但只要全市上下同心同德、砥砺奋进，就没有克服不了的困难，就没有战胜不了的风险。让我们在市委的坚强领导下，以更加饱满的热情、更加昂扬的斗志、更加务实的作风，凝心聚力，共克时艰，为谱写好中国梦的赤峰篇作出新的更大贡献！
